# A hybrid register and questionnaire study of Covid-19 and post-acute sick leave in Denmark

**DOI:** 10.1038/s41467-023-42048-1

**Published:** 2023-10-07

**Authors:** Elisabeth O’Regan, Ingrid Bech Svalgaard, Anna Irene Vedel Sørensen, Lampros Spiliopoulos, Peter Bager, Nete Munk Nielsen, Jørgen Vinsløv Hansen, Anders Koch, Steen Ethelberg, Anders Hviid

**Affiliations:** 1 Department of Epidemiology Research, Statens Serum Institut, 2300 Copenhagen S, Denmark; 2 Infectious Disease Epidemiology and Prevention, Statens Serum Institut, 2300 Copenhagen S, Denmark; 3 Focused Research Unit in Neurology, Department of Neurology, Hospital of Southern Jutland, University of Southern Denmark, 6200 Aabenraa, Denmark; 4 Department of Public Health, Global Health Section, University of Copenhagen, Copenhagen, Denmark; 5 Department of Infectious Diseases, Rigshospitalet University Hospital, 2100 Copenhagen Ø, Denmark; 6 Pharmacovigilance Research Center, Department of Drug Design and Pharmacology, University of Copenhagen, 2100 Copenhagen Ø, Denmark

**Keywords:** Epidemiology, Risk factors, Outcomes research, SARS-CoV-2

## Abstract

Post-acute sick leave is an underexplored indicator of the societal burden of SARS-CoV-2. Here,  we report findings about self-reported sick leave and risk factors thereof from a hybrid survey and register study, which include 37,482 RT-PCR confirmed SARS-CoV-2 cases and 51,336 test-negative controls who were tested during the index- and alpha-dominant waves. We observe that an additional 33 individuals per 1000 took substantial sick leave following acute infection compared to persons with no known history of infection, where substantial sick leave is defined as >1 month of sick leave within the period 1–9 months after the RT-PCR test date. Being female, 50–65 years, or having certain pre-existing health conditions such as obesity, chronic lung diseases, and fibromyalgia each increase risk for taking substantial sick leave. Altogether, these results may help motivate improved diagnostic and treatment options for persons living with post-Covid conditions.

## Abstract (web summary)

Authors utilise a questionnaire-based approach to survey self-reported, post-acute sick leave and risk factors in cases of SARS-CoV-2.

## Introduction

In May 2023, the World Health Organization (WHO) Director-General determined that SARS-CoV-2 no longer constitutes a public health emergency of international concern^1^. As societies have gradually adjusted to living with SARS-CoV-2, many research efforts have shifted from rapid emergency assessments to retrospective analyses of the health and economic burden countries have sustained. In particular, quantifying the societal impact of post-Covid conditions has been difficult, as the diagnosis can be hard to reach due to symptom variability^2–5^, and it is challenging to disentangle the post-acute effects of SARS-CoV-2 infection from symptoms of pre-existing health conditions^6^. Furthermore, it remains unclear how post-acute effects of SARS-CoV-2 infection affect daily living and quality of life at the societal level, how this differs by variant and vaccination status^6^, and which groups have been most at-risk of experiencing prolonged illness^7–10^.

One underexplored indicator of the societal impact of SARS-CoV-2 is post-acute sick leave. In Denmark, an earlier study on post-acute symptoms identified sick leave as a potential indicator of the burden of post-Covid conditions, where both full- and part-time sick leave were more frequent among test-positives for SARS-CoV-2 compared to test-negatives^11^. Still, the extent to which these symptoms translate to working ability remains understudied, and current literature on the subject has largely lacked control groups with no history of SARS-CoV-2 infection^4,12–16^.

Using post-acute sick leave to explore the societal burden of SARS-CoV-2 and to study individual risk factors can help broaden understanding of the prolonged consequences of infection. Quantifying sick leave taken during previous waves of the pandemic is also necessary to gain insight into how the prolonged consequences of infection evolve in the context of vaccination, different variants, and reinfection. This information is needed to build capacity for future outbreaks and to develop better-targeted treatment plans for individuals living with post-acute symptoms.

The aims of this study were (1) to evaluate the association between SARS-CoV-2 infection and post-acute sick leave and (2) to explore the possible impact of age, sex, and pre-existing health conditions on this association. Specifically, we examined post-acute sick leave following infections that took place during the index- and alpha-dominant periods in Denmark by comparing persons infected with SARS-CoV-2 to persons with no known history of SARS-CoV-2 infection.

In this paper, we observe that an additional 33 individuals per 1000 took substantial sick leave following acute infection compared to persons with no known history of infection. We furthermore see that being female, 50–65 years, or having certain pre-existing health conditions such as obesity, chronic lung diseases, and fibromyalgia each increased risk for taking substantial sick leave.

## Results

### Overview of study population

**Fig. 1:**
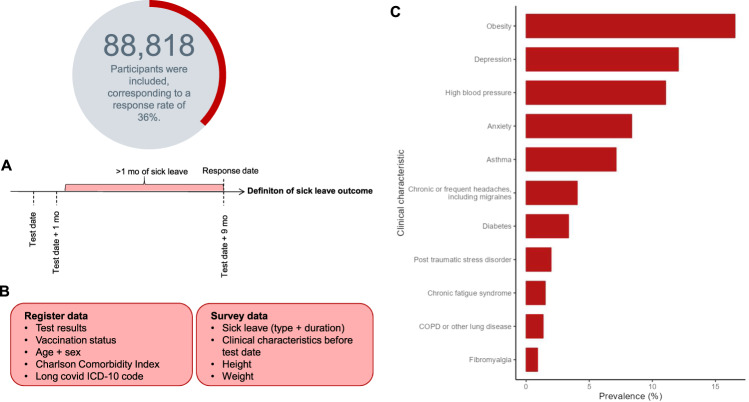
Overview of study population and study variables. *N* = 88,818 participants ages 15–65 were included (response rate = 36%). **A** Definition of the sick leave outcome, substantial sick leave. Substantial sick leave was defined as >1 month of sick leave in the period 1–9 months after the test. **B** Overview of study variables pulled from national register data and survey data. **C** Prevalence of each clinical characteristic/pre-existing condition in the total study sample, including both test-positives and -negatives for SARS-CoV-2.

Out of 294,035 invited, a total of 106,917 persons fully completed a questionnaire nine months after testing positive or negative for SARS-CoV-2 (response rate 36.4%). After all exclusion criteria were applied, the study population consisted of 88,818 individuals, of which 37,482 had had SARS-CoV-2 infection confirmed with a positive reverse transcription polymerase chain reaction (RT-PCR) test (Fig. S1). Of all participants, 64.3% were female, and the mean age was 45 years (SD 13.8) (Table 1). Less than 1% of participants had received one or more doses of a vaccine against SARS-CoV-2. Based on self-reported height and weight, 16.6% of the study population was identified as obese. Apart from obesity, the most frequent preexisting clinical characteristics were depression, high blood pressure, and anxiety (Table 1 and Fig. 1C). Respondents were more likely to be female, older (50–65 years), and have a higher Charlson Comorbidity Index than non-respondents (Table S4).

**Table 1:** Characteristics of 88,818 study participants who obtained a positive or negative PCR test for SARS-CoV-2

Characteristics	Tested negative	Tested positive	*p* value
	(*N* = 51,336)	(*N* = 37,482)	
Sex			
Female	34,085 (66.4%)	23,002 (61.4%)	≤0.001
Male	17,251 (33.6%)	14,480 (38.6%)	
Age			
Mean (SD)	45.8 (13.7)	43.6 (13.9)	≤0.001
Median [Min, Max]	49.0 [15.0, 65.0]	46.0 [15.0, 65.0]	
Charlson Comorbidity Index			
0	45,984 (89.6%)	33,988 (90.7%)	≤0.001
1	2854 (5.6%)	1967 (5.2%)	
2	1926 (3.8%)	1144 (3.1%)	
3 or more	572 (1.1%)	383 (1.0%)	
Educational level (highest)			
Higher education (>5 years, MSc, PhD)	8938 (17.4%)	6799 (18.1%)	≤0.001
Higher education (2–4 years, BSc)	17,237 (33.6%)	12,147 (32.4%)	
Higher education (1–2 years, vocational academy)	5999 (11.7%)	4067 (10.9%)	
Primary or elementary school (9th–10th grade)	4267 (8.3%)	2991 (8.0%)	
General secondary or vocational secondary education	5127 (10.0%)	4711 (12.6%)	
Vocational training	8490 (16.5%)	5755 (15.4%)	
Don’t know	1278 (2.5%)	1011 (2.7%)	
Healthcare worker			
No	42,662 (83.1%)	32,284 (86.1%)	≤0.001
Yes	8674 (16.9%)	5198 (13.9%)	
Obesity			
No	38,696 (75.4%)	28,599 (76.3%)	≤0.001
Unknown	4143 (8.1%)	2680 (7.2%)	
Yes	8497 (16.6%)	6203 (16.5%)	
Fibromyalgia			
No	50,339 (98.1%)	36,783 (98.1%)	0.695
Before test	474 (0.9%)	329 (0.9%)	
Chronic fatigue syndrome			
No	49,782 (97.0%)	35,633 (95.1%)	≤0.001
Before test	863 (1.7%)	482 (1.3%)	
Anxiety			
No	45,847 (89.3%)	33,301 (88.8%)	≤0.001
Before test	4431 (8.6%)	2994 (8.0%)	
Depression			
No	43,759 (85.2%)	32,068 (85.6%)	≤0.001
Before test	6466 (12.6%)	4249 (11.3%)	
PTSD			
No	49,686 (96.8%)	36,297 (96.8%)	0.48
Before test	1037 (2.0%)	720 (1.9%)	
Asthma			
No	47,840 (93.2%)	34,643 (92.4%)	≤0.001
Before test	3496 (6.8%)	2839 (7.6%)	
Diabetes			
No	49,583 (96.6%)	36,255 (96.7%)	0.256
Before test	1753 (3.4%)	1227 (3.3%)	
High blood pressure			
No	45,412 (88.5%)	33,589 (89.6%)	≤0.001
Before test	5924 (11.5%)	3893 (10.4%)	
COPD or other lung disease			
No	50,565 (98.5%)	37,056 (98.9%)	≤0.001
Before test	771 (1.5%)	426 (1.1%)	
Chronic or frequent headaches or migraines			
No	49,212 (95.9%)	36,013 (96.1%)	0.107
Before test	2124 (4.1%)	1469 (3.9%)	

*p* Values for the association between test status and the survey participant characteristics were estimated using Student’s *t* test for continuous variables and Pearson’s Chi-squared test for categorical variables. No adjustment for multiple comparisons were made.

*PTSD* post-traumatic stress disorder, *COPD* chronic obstructive pulmonary disease.

### Prevalence of substantial sick leave

**Fig. 2:**
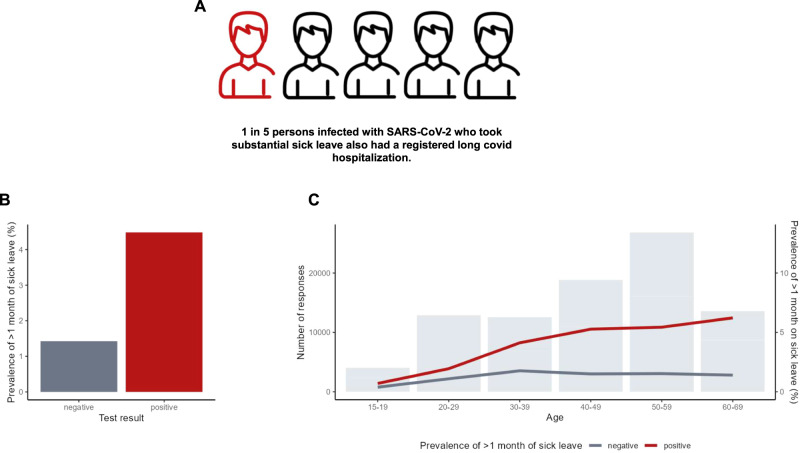
Prevalence of substantial sick leave, defined as >1 month of sick leave in the period 1–9 months after the test date. *N* = 88,818 participants ages 15–65 were included (response rate = 36%). N_positive_ = 51,336, N_negative_ = 37,482. **A** 21.1% of persons infected with SARS-CoV-2 who took substantial sick leave also had a registered long Covid hospitalization. Long Covid hospitalization was defined as having a registered International Classification of Diseases 10th Revision (ICD-10 code B948A) in the period 1–9 months after the test date and having no history of this diagnosis within the year prior to the test date. **B** Unadjusted prevalence of substantial sick leave by SARS-CoV-2 PCR test result. **C** Number of responses and prevalence of substantial sick leave by age group and PCR test result.

The prevalence of substantial sick leave was 1.4% among test-negatives compared to 4.5% among test-positives. Among test-negatives only, the prevalence of substantial sick leave was similar across age groups. Conversely, for test-positives, the prevalence of substantial sick leave increased with age (Fig. 2C). Notably, out of all test-positives who took substantial sick leave, 21.1% (*N* = 354) individuals had received a hospital-registered diagnosis with sequelae of SARS-CoV-2 (ICD-10 code B948A) (Fig. 2A). However, out of all the test-positives who did *not* take substantial sick leave, only 1.6% (*N* = 556) also had a hospital-registered diagnosis with sequelae of SARS-CoV-2.

**Fig. 3:**
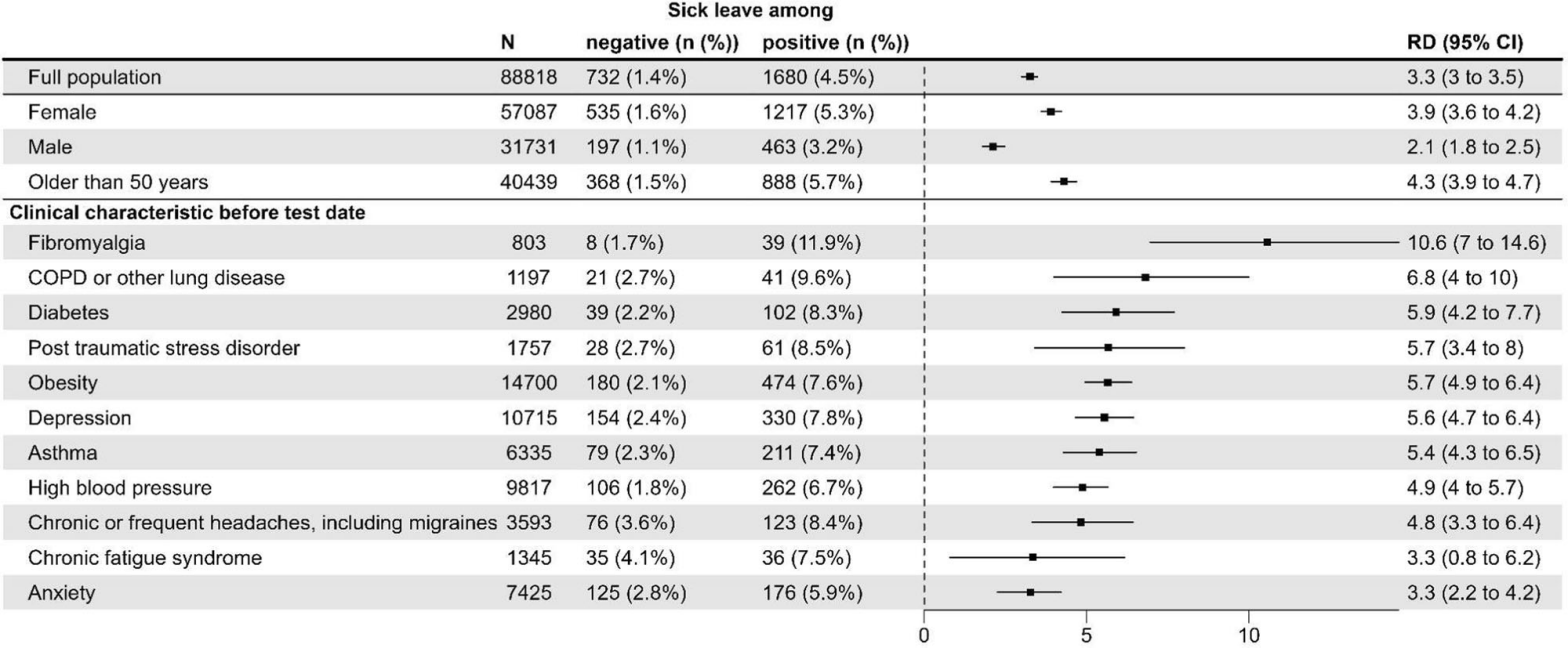
Risk differences (RDs) and 95% confidence intervals (CIs) for full-time substantial sick leave taken one to nine months after the test date between SARS-CoV-2 test-positives and test-negatives for the total study population and possible long Covid risk groups. RDs are adjusted for age, sex, Charlson Comorbidity Index, educational level, and select pre-existing conditions (chronic diseases). *N* = 88,818 participants ages 15–65 years were included (response rate = 36%). *N*_positive_ = 51,336, *N*_negative_ = 37,482. RDs are expressed in percentage points.

In view of possible risk groups identified in previous literature^9,12^^[,13,17^, we stratified on sex, middle to older age (50–65 years), and pre-existing health conditions. Across strata, the prevalence of substantial sick leave was higher among test-positives compared to test-negatives. Among persons with pre-existing health conditions, the baseline prevalence of substantial sick leave, i.e., among test-negatives, was higher than for the test-negative general population (1.4%), and the highest background prevalence was among individuals with chronic fatigue syndrome (4.1%) (Fig. 3).

### Risk differences (RDs) for substantial sick leave

In the 8 months following acute infection with SARS-CoV-2, and during the index- and alpha-dominant waves, persons infected with SARS-CoV-2 had a higher risk (RD 3.3, 95% confidence interval (CI) 3.0–3.5) of taking substantial sick leave after their acute infection (>1 month of sick leave within 1–9 months after the test date) compared to test-negatives with no known history of SARS-CoV-2 infection (Fig. 3). Changing the definition of substantial sick leave by increasing the duration resulted in attenuation of the RD (e.g., RD = 0.5, 95% CI 0.4–0.6) for substantial sick leave defined as at least 6 months (Fig. S3). A distributional plot underlying substantial sick leave by age (<50 years, ≥50 years) and sex is available in Fig. S4.

RDs were greater for females (RD 3.9, 95% CI 3.6–4.2) than males (RD 2.1, 95% CI 1.8–2.5). For middle-older age (50–65 years) and all pre-existing health conditions except chronic fatigue syndrome and anxiety, estimated RDs were higher than that in the general population. The largest RDs were observed for persons with fibromyalgia (RD 10.6, 95% CI 7–14.6), COPD or other lung disease (RD 6.8, 95% CI 4–10) and diabetes (RD 5.9, 95% CI 4.2–7.7). Obesity, the most frequent clinical characteristic, had a larger RD (RD 5.7, 95% CI 4.9–6.4) than that in the general population (RD 3.3, 95% CI 3–3.5) (Fig. 3). RDs by education level showed that individuals with a “higher education of 2–4 years (e.g., nurse, preschool teacher, bachelor of engineering)” had a larger RD (RD 3.8, 95% CI 3.4–4.2) than that in individuals with a “higher education of ≥5 years (e.g., master’s degree or PhD)” (RD 1.9, 95% CI 1.4–2.4) (Table S2). Healthcare workers (*N* = 13,872) also had a larger RD (RD 4.6, 95% CI 3.9–5.3) than the general population (RD 3.3, 95% CI 3.0–3.5).

### RDs for part-time sick leave

Furthermore, persons infected with SARS-CoV-2 had a higher risk (RD 2.1, 95% CI 2.0–2.3) of substantial part-time sick leave compared to test-negatives. Similar results when stratifying on sex were observed for both part-time and full-time sick leave. However, with regards to individuals 50–65 years, a greater difference in RD was noted for full-time sick leave than for part-time sick leave. For full-time sick leave, this age group had a higher RD (RD 4.3, 95% CI 3.9–4.7) than that of the full population (RD 3.3, 95% CI 3.0–3.5). For part-time sick leave, individuals 50–65 years had a similar risk (RD 2.3, 95% CI 2.1–2.6) compared to that of the full study population (RD 2.1, 95% CI 2.0–2.3) (Fig. S2).

## Discussion

In this study, we explored the burden of post-acute sick leave following SARS-CoV-2 infection. First, we found that individuals infected with SARS-CoV-2 during the index- and alpha-dominant waves had a significantly higher risk of substantial sick leave than those who had no history of infection (3.3 percentage point increase in risk), where substantial sick leave was defined as >1 month of self-reported sick leave within the period 1–9 months after the test. Second, we observed that females, persons 50–65 years, and persons with pre-existing fibromyalgia, obesity, and lung diseases had a markedly higher risk of post-acute, substantial sick leave than the general population. Finally, 21.1% of the test-positives who took substantial sick leave also had a hospital-registered diagnosis of sequelae of SARS-CoV-2 (long Covid). Altogether, these findings suggest that infections which took place during the index- and alpha-dominant waves posed a considerable burden to society in the form of post-acute sick leave.

Other work has examined sick leave in persons previously infected with SARS-CoV-2; however, differences in risk measures, timing of measurements, national testing strategies, and definitions of sick leave vary between studies and should be carefully considered. A Danish register-based cohort study with 7466 participants examined return-to-work following first-time infections occurring between January and May 2020, where 81.9% returned to work within four weeks of their first positive SARS-CoV-2 test^18^. Although this study did not have a test-negative control group, the authors examined the cumulative incidence of return to work between patients admitted to hospitals with SARS-CoV-2 and patients admitted with influenza, which suggested that patients with SARS-CoV-2 had a reduced chance of returning to work compared to patients admitted with influenza. Additionally, a Danish cross-sectional study which examined the influence of long Covid on activities of daily living among 448 long Covid patients reported that 56% needed sick leave and 94% were referred to rehabilitation^16^. In a German register-based study with 30,950 individuals diagnosed with SARS-CoV-2, a reported 5.8% of individuals took more than four weeks of sick leave between March 2020 and February 2021^12^. These studies, together with ours, point to sick leave as one indicator of the post-acute personal and societal burden of SARS-CoV-2. Intriguingly, what may be more telling is the heterogeneity of sick leave following SARS-CoV-2 for the purpose of identifying possible long Covid risk groups.

Female sex has been identified as a possible risk factor for long Covid^6^, and some studies have indicated that females needed longer sick leave following infection with SARS-CoV-2 than their male counterparts^12,18^. Others have pointed to interactions between age and sex, e.g., less sick leave in infected females ages 20–44 compared to infected females ages 45–70 years^19^. Additionally, a Swedish registry-based cohort study reported that people with recurrent sick leave were older, more often female, and more likely to have been on sick leave prior to the pandemic^14^. In our study, we observed that the RD for substantial sick leave between SARS-CoV-2 test-positives and -negatives was greater for females (RD 3.9, 95% CI 3.6–4.2) than for males (RD 2.1, 95% CI 1.8–2.5), and that females as well as persons 50–65 years had a slightly higher prevalence of sick leave compared to the general population, irrespective of test status. Importantly, severity of SARS-CoV-2 infection is associated with increased age, pre-existing medical conditions, and male sex^20^, and severity of infection has been associated with long Covid^6^. It is therefore unclear as to why females appear to be a risk group for substantial sick leave following acute SARS-CoV-2 infection but males are not. Further research examining age-sex interactions and the impact of multimorbidity on post-acute outcomes are needed^21^.

A German register-based study by Jacob et al.^12^ also explored a range of possible risk factors for sick leave, some of which overlapped with risk factors that we explored. As in our study, the authors also found that diabetes and high blood pressure were positively and significantly associated with long-term sick leave. However, in contrast to our study findings, the authors did not find obesity and asthma to increase the risk of long-term sick leave. Furthermore, the authors reported that anxiety and somatoform disorders were not associated with the risk of longer-term sick leave. Similarly, in our study, neither anxiety nor chronic fatigue syndrome, which is categorized as a somatoform disorder by some, increased the risk of substantial sick leave; however, new-onset chronic fatigue syndrome is a known complication of long Covid^6^.

Fibromyalgia, COPD/other chronic lung diseases, and obesity have also been cited as possible risk factors for long Covid in other work. A US study (preprint) (*N* = 89,843) found that long Covid patients were more likely to have a history of fibromyalgia (OR 2.3, 95% CI 1.3–3.8) and chronic pulmonary lung disease (OR 1.9, 95% CI 1.5–2.6) compared to matched test-positive controls without long Covid^22^. Additionally, obesity has been identified as a risk factor for severe acute SARS-CoV-2 infection^17^, and inpatient care following infection has been suggested to predict longer sick leave^13^.

Finally, individuals’ line of work can impact the need for post-acute sick leave following SARS-CoV-2 infection. Although we were not able to examine most individuals’ professions since we did not have access to occupational data at the date they responded to the survey, we did observe that individuals with a higher education of 2–4 years had a larger RD of taking substantial sick leave compared to individuals with a postgraduate education. These differences might be attributed to job adaptability to work-from-home and self-pacing, i.e., persons with a post-graduate education may work desk jobs whereas persons with a higher education of 2-4 years (e.g., nurse, preschool teacher, bachelor of engineering) may need to be on-site. Similar findings were reported in a Norwegian study, which estimated the industry-specific impact of the Omicron wave on sick leave compared to corresponding months from 2017 to 2020^23^. Persons within the food and accommodation industry had the highest increase in sick leave (4.4 percentage points increase, 95% CI 4.3–4.5), suggesting that individuals with jobs not-suited for work from home required more sick leave.

While we observed that healthcare workers had a greater RD for post-acute substantial sick leave than the general population, little is known about the post-acute burden of SARS-CoV-2 infection in healthcare workers. Healthcare workers are at an elevated risk of acute SARS-CoV-2 infection compared to the general population, and as such, the burden of long Covid, and consequently the burden of post-acute sick leave may be large^24^.

The present study describes population-level data on post-acute sick leave as a possible consequence of SARS-CoV-2 infection. In contrast to most existing literature on the subject of post-acute burden following SARS-CoV-2 infection and working ability, this study used a test-negative control group, allowing us to consider background prevalence of sick leave for the study population and among possible risk groups, including people with various conditions which preceded their RT-PCR test. Furthermore, our sick leave outcome variable captures fluctuating illness, which is useful given what is known about the irregularity of long Covid symptoms.

We consider the main limitations of this study to be its self-reporting nature and potential participation and recall bias. One shortcoming of the self-reported sick leave variable is that the duration of sick leave cannot be directly attributed to long Covid symptoms, as the questionnaire asked about sick leave in general rather than sick leave associated with SARS-CoV-2 infection. A possible alternative to studying self-reported sick leave was using register-based sick leave benefits. However, the prevalence of register-based sick leave benefits was previously examined for infections occurring between January and May 2020 in Denmark^18^, where the authors used data from the Danish Register for Evaluation of Marginalization (DREAM). DREAM primarily covers sick leave benefits which are granted after 30 days of sickness absence. A disadvantage of using that register data is that it largely excludes shorter periods of absence^25^ and thus is not well-suited for capturing fluctuating illness as a possible consequence of long Covid.

Participation bias may have occurred, where individuals living with poor health or long Covid symptoms may have taken more interest in participating. Alternatively, some individuals living with long Covid symptoms may have felt too poorly to participate. In addition, the retrospective study design is vulnerable to recall bias, where some participants may not remember how much sick leave they took over the 9 months following their test. We sought to reduce this by multiple choice between pre-defined sick leave durations instead of using free-text. While the RD was attenuated for increasing durations of sick leave as expected, the RD for at least 6 months (RD 0.5, 95% CI 0.4–0.6) is still quite striking considering the large number of infected globally. Importantly, our results capture index- and alpha-variant infections which largely occurred before SARS-CoV-2 vaccine rollout, and we cannot exclude that the absolute magnitude of our results are attenuated by vaccinations and the omicron variant. Finally, post-acute sick leave is not a suitable measure for retired or non-working individuals, and as such, other indicators of the post-acute burden of SARS-CoV-2 infection are needed.

The post-acute burden of SARS-CoV-2 infection is a concerning, contemporary public health issue with many unanswered questions, particularly regarding manifestations of symptom burden and risk factors. This study provides much-needed information on post-acute sick leave following SARS-CoV-2 infection in a general population and the risk of post-acute sick leave for people with comorbid conditions. The results from this study may be particularly useful to public health stakeholders in guiding evidence-based decisions concerning targeted preventative strategies. Further long Covid/post-acute research and funding initiatives are critical, particularly to increase knowledge of immunopathogenic mechanisms, phenotypes, risk factors, and the impact of different (sub)variants and SARS-CoV-2 vaccines.

In sum, among individuals infected with SARS-CoV-2 during the index- and alpha-dominant waves, an additional 33 individuals per 1000 took substantial sick leave within 1–9 months following acute infection compared to persons with no known infection. Females, individuals aged 50–65 years, and individuals with pre-existing health conditions such as fibromyalgia, chronic lung diseases, and obesity were markedly affected. This study may be used to help inform the healthcare and research workforce of the post-acute impact of SARS-CoV-2 on working ability and to motivate improved diagnostic and treatment options for persons living with post-Covid conditions.

## Methods

### Ethical approval

This study was performed as a surveillance study as part of the governmental institution Statens Serum Institut’s (SSI) advisory tasks for the Danish Ministry of Health. SSI’s purpose is to monitor and fight the spread of disease in accordance with section 222 of the Danish Health Act. According to Danish law, national surveillance activities carried out by SSI do not require approval from an ethics committee.

Participation in the study was voluntary. The invitation letter to participants contained information about their rights under the Danish General Data Protection Regulation (rights to access data, rectification, deletion, restriction of processing and objection). After reading this information, it was considered informed consent if participants agreed and clicked on the link to fill in the questionnaires.

### Study context

#### Denmark’s universal SARS-CoV-2 testing strategy

In this study, it is important to consider Denmark’s national SARS-CoV-2 testing strategy and how this has facilitated the conduct of population-level studies with test-negative control groups, particularly before Omicron and the availability of home rapid antigen tests. In Denmark, universal testing for SARS-CoV-2 was implemented from the end of May 2020 and continued throughout the testing period under study from November 2020 to February 2021. Reverse transcription polymerase chain reaction (RT-PCR) tests were available and accessible for all adults free of charge and independent of the indication for acquiring a test^26^. Additionally, persons admitted to hospitals were tested for SARS-CoV-2. During the testing period under study between November 2020 and February 2021 (index- and alpha-dominant waves), the weekly PCR test incidence in Denmark ranged from 7900 tests to 14,700 tests per 100,000 inhabitants (mean of 10,800 tests per 100,000 inhabitants)^27^. Since February 2020, all RT-PCR test results have been registered in the Danish microbiology database (MiBa)^28^, and variant dominance has been determined by extensive whole genome sequencing^29^.

#### Sick leave in Denmark

Most employees in Denmark are covered under the Act on Salaried Employees and are thus entitled to full pay during sick leave^30^. Employers typically cover the first four weeks of sick leave, after which municipalities bear the costs.

### Study design

In this cohort study, we merged nationwide survey- and register data. In Denmark, all residents are assigned a unique identifier (the CPR-number) in the Danish Civil Registration System, and this number is commonly used to link individual-level data from varying data sources. A summary of the characteristics of our study population and which variables we used from survey responses and register data are presented in Fig. 1.

#### The EFTER-COVID survey

To investigate self-reported sick leave after infection with SARS-CoV-2, we used data from a nationwide Danish survey, EFTER-COVID (AFTER COVID). This survey was launched in August 2021 to investigate the general health of the Danish population during the pandemic with a particular focus on long Covid. Based on RT-PCR test results recorded in MiBa, individuals were sent an invitation to participate in the study via the national digital mail system. This system (“e-Boks”) enables secure electronic communication with public authorities and is used by over 90% of all Danish residents aged ≥15 years^31^. EFTER-COVID survey data were collected using Danish- or English-language web-based questionnaires created in SurveyXact^32^, which could be filled out using a PC, tablet, or smartphone. An English translation of the questionnaire is available in Supplementary Information of a previous EFTER-COVID study^11^.

All Danish residents who had an e-Boks account and obtained a first positive RT-PCR test result for SARS-CoV-2 registered in MiBa during the period from November 2020 to February 2021 were invited to participate in the EFTER-COVID survey. Additionally, test-negative controls were randomly selected using incidence density sampling on the test date with a ratio of 2:3 between test-positive and -negative persons. This ratio was chosen to compensate for a lower expected response rate among controls compared to cases. Importantly, the test-negative controls did not have a registered positive test result in MiBa at any time point prior to receiving or responding to the questionnaire, which we could reassure due to the extensive national testing strategy. In this study, we included participants who responded to a retrospective questionnaire 9 months after their test date. Other studies which have used the EFTER-COVID survey data^11,33,34^ include participants who responded at other points in time after testing and thus reflect different subsets of EFTER-COVID data, which contain more than 840,000 survey participants^35^.

#### Data sources

Using the CPR-number, we enriched the EFTER-COVID questionnaire data with register-based information on age and sex, SARS-CoV-2 vaccinations from the Danish Vaccination Register (DDV)^36^, SARS-CoV-2 test results and (re)infection history registered in MiBa, healthcare workers from work authorization from the Danish Register of Healthcare Professionals^37^, and comorbidities from the Danish National Patient Register (DNPR)^38^ five years prior to each participant’s test date.

The questionnaire included questions about baseline characteristics of the participant, including height, weight, education, smoking habits, alcohol consumption, and health conditions preceding the individual’s test date. In addition, participants were asked about the amount of full- or part-time sick leave they took between their test date (indicated in the questionnaire), and the day they responded to the survey. Test-negatives were asked whether they suspected ever having had SARS-CoV-2, e.g., if they had received a seropositive test result. All questions required a response to complete the questionnaire, except for height, weight, smoking and alcohol consumption.

From the DNPR, we obtained information on in- and outpatient diagnoses coded using the 10^th^ revision of the International Statistical Classification of Diseases and Related Problems (ICD-10), which enabled the calculation of Charlson Comorbidity Index. We also extracted information on the long Covid ICD-10 code (ICD-10 code B948A) from the DNPR. A complete description of how we categorized these variables can be found in Table S1.

#### Exclusion criteria

Participants who did not complete the questionnaire were excluded. Furthermore, we did not include individuals who indicated that they believed they previously had SARS-CoV-2 due to receiving a seropositive result for SARS-CoV-2. Participants who were >65 years were also excluded due to retirement age, where age was calculated on the test date. See Fig. S1 for a detailed flowchart of our inclusion and exclusion criteria.

#### Outcomes

All participants, regardless of test status, were asked whether they took sick leave around the time of their test date or at any time point after the test. Individuals who responded “yes” to taking sick leave more than four weeks after the test were then asked whether their sick leave was full-time, part-time, or both and for how long they were on full- and/or part-time sick leave.

A binary outcome variable was defined as having taken “*no or* ≤*4 weeks of full-time sick leave* >*4 weeks after the test date*” or “*>4 weeks of full-time sick leave >4 weeks after the test date*”. The latter was treated as indicative of substantial sick leave, i.e., at least one full month of sick leave was taken over an eight-month period one month after the test. An identical outcome was defined for part-time sick leave.

We chose to examine sick leave from one month after the test to capture sick leave after the acute infection period among test-positives. The self-reported sick leave was not necessarily taken consecutively such that it could capture multiple periods of sick leave due to fluctuating symptoms.

Participants who reported taking only sick leave during the week up to the test and up to four weeks after the test where included in the category “*no or* <*4 weeks of sick leave >4 weeks after the test date*”. For an illustration of how we defined our sick leave outcome, see Fig. 1A.

### Statistical methods

The prevalence of substantial sick leave among test-positives and test-negatives were compared using RDs, which give the difference between the risk of an outcome in the exposed group and the unexposed group. Parametric g-computation on logistic regression^39^ was used to estimates RDs with 95% CIs obtained using bootstrap random resampling with 1000 iterations comparing test-positive and test-negative individuals with adjustments for age, sex, Charlson Comorbidity Index, self-reported chronic illness (diabetes, asthma, high blood pressure, chronic obstructive pulmonary disease (COPD) or other chronic lung disease, chronic or frequent headaches/migraines), and education level. RDs for the risk of substantial sick leave are expressed in percentage points. *p* Values for the association between test status and the survey participant characteristics were estimated using Student’s *t* test for continuous variables and Pearson’s Chi-squared test for categorical variables.

To investigate possible risk groups for substantial sick leave following infection with SARS-CoV-2, we conducted analyses on sub-populations defined by possible risk factors. These risk factors were defined apriori based on available variables in the survey. These included middle to older age (categorized as 50–65 years), female sex, obesity, diabetes, asthma, high blood pressure, COPD or other chronic lung disease, chronic or frequent headaches/migraines, and the following health conditions diagnosed by a medical doctor before the test: depression, anxiety, post-traumatic stress disorder, chronic fatigue syndrome, and fibromyalgia. In addition, we restricted analyses by educational level and healthcare workers. RDs with 95% CIs were estimated as described above.

All statistical analyses were carried out in R version 4.2.2^40^. The R-packages “riskCommunicator” (v1.0.1)^41^ was used for modeling and “forestploter” (v0.2.3)^42^ for data visualization (forest plots).

### Reporting summary

Further information on research design is available in the Nature Portfolio Reporting Summary linked to this article.

### Supplementary information

Supplementary Information

Peer Review File

Reporting Summary

## Data Availability

The datasets used in this study comprise sensitive, individual-level information from completed questionnaires and national register data. According to the Danish data protection legislation, the authors are not permitted to share these sensitive data directly upon request. However, the data are available for research purposes upon request to the Danish Health Authority (register data, email: kontakt@sundhedsdata.dk) and Statens Serum Institut (questionnaire data, email: aii@ssi.dk), as well as within the framework of the Danish data protection legislation and any required permission from authorities. Data request processing can take an expected 3–6 months.

## Code availability

The underlying code for this study are not publicly available.
