# Supplementary material for: A hybrid register and questionnaire study of Covid-19 and post-acute sick leave in Denmark
Source: Nat Commun. 2023 Oct 7;14:6266. doi: 10.1038/s41467-023-42048-1 (PMC10560282; doi:10.1038/s41467-023-42048-1)
Supplement: Supplementary file 1 — Supplementary Information [file 41467_2023_42048_MOESM1_ESM.pdf]

Table of Contents: Supplementary Information

Figure S1: Flowchart of study population. 2

Table S1: Overview of study variables and data sources 3

Figure S2: Risk differences (RDs) and 95% confidence intervals (CIs) for part-time substantial sick leave one to nine months after test date between SARS-CoV-2 test-positives and negatives 6

Figure S3: Risk differences (RDs) and 95% confidence intervals (CIs) for sick leave by differing duration. RDs are adjusted for age, sex, Charlson Comorbidity Index, educational level, and select pre-existing conditions (chronic diseases) 7

Figure S4: A distributional plot of the underlying self-reported sick leave by age (<50 years, ≥50 years) and sex. 8

Table S2: Risk differences (RDs) and 95% confidence intervals (CI) for substantial sick leave taken one to nine months after the test date between SARS-CoV-2 test-negatives and positives for the total study population, stratified on educational level. 9

Table S3: Characteristics of test-positive population divided into subcategories based on substantial sick leave and long covid hospitalization. 10

Table S4: Background characteristics for respondents, partial-respondents, and non-respondents 12

Figure S1: Flowchart of study population.

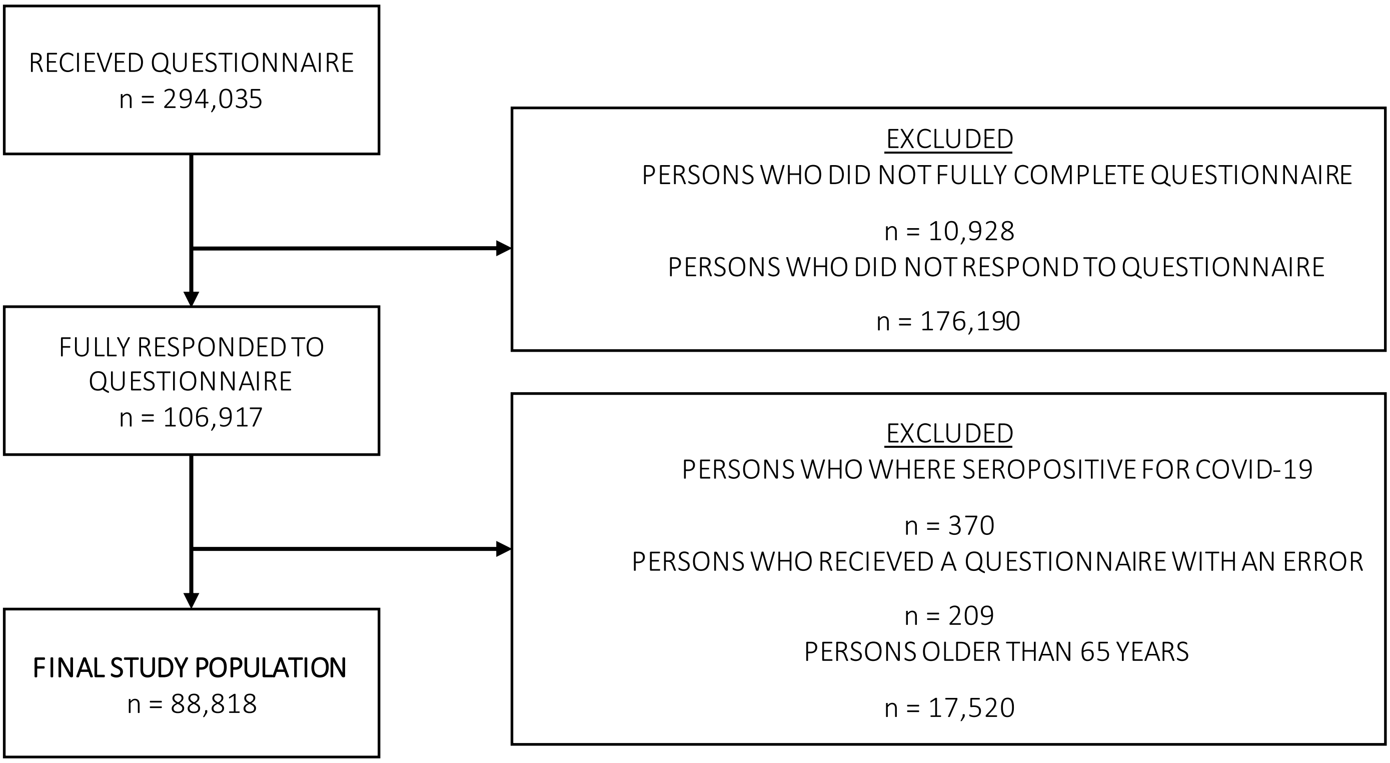

Overview of how study participants were included and excluded.

Table S1: Overview of study variables and data sources

Exposure	Description	Data type	Categorization	Data source
SARS-CoV-2 infection	Registered RT-PCR test result.	Categorical variable.	"Positive" (exposure)<br>"Negative" (reference)	MiBa
**Outcomes**	**Description**	**Data type**	**Categorization**	**Data source**
Sick leave	Self-reported.	Categorical variable.	"No or ≤4 weeks of sick leave >4 weeks after the test date"<br>">4 weeks of sick leave >4 weeks after the test date"	EFTER COVID
**Covariates**	**Description**	**Data type**	**Categorization**	**Data source**
Age	Registered.	Categorical variable.	"15-19 years"<br>"20-29 years"<br>"30-39 years"<br>"50-59 years"<br>"60-65years"	CPR
Sex	Registered.	Categorical variable.	"Male"<br>"Female"	CPR
Charlson Comorbidity Index	Self-reported. Based on Charlson Comorbidity Index.	Categorical variable.	"0"<br>"1"<br>"2"<br>"≥3"	EFTER COVID
Vaccination status	Registered.	Categorical variable.	"Primary course >2 weeks before test date"<br>"0 doses"	DNPR
Smoking	Self-reported.	Categorical variable.	"Current smoker"<br>"Previous smoker"<br>"Electronic cigarette smoker"<br>"Never smoker"	EFTER COVID
Alcohol consumption	Self-reported.	Categorical variable.	"No or moderate; ≤10 units of pure alcohol per week"<br>"Heavy; >10 units of 15ml of pure alcohol per week"	EFTER COVID
Fibromyalgia	Self-reported.	Categorical variable.	"Yes, before test"<br>"No"	EFTER COVID

COPD/other chronic lung disease	Self-reported.	Categorical variable.	“Yes, before test”<br>“No”	EFTER COVID
Diabetes	Self-reported.	Categorical variable.	“Yes, before test”<br>“No”	EFTER COVID
PTSD	Self-reported.	Categorical variable.	“Yes, before test”<br>“No”	EFTER COVID
Depression	Self-reported.	Categorical variable.	“Yes, before test”<br>“No”	EFTER COVID
Anxiety	Self-reported.	Categorical variable.	“Yes, before test”<br>“No”	EFTER COVID
Asthma	Self-reported.	Categorical variable.	“Yes, before test”<br>“No”	EFTER COVID
Hypertension	Self-reported.	Categorical variable.	“Yes, before test”<br>“No”	EFTER COVID
Chronic headaches	Self-reported.	Categorical variable.	“Yes, before test”<br>“No”	EFTER COVID
Chronic fatigue syndrome	Self-reported.	Categorical variable.	“Yes, before test”<br>“No”	EFTER COVID
Obesity	Height and weight were self-reported. Obesity was based on BMI*.	Categorical variable.	“Yes”<br>“No”	EFTER COVID
Education	Self-reported.	Categorical variable.	“Higher education (>5 years, MSc, PhD)”<br>“Higher education (2-4 years, BSc)”<br>“Higher education (1-2 years, vocational academy)”<br>“General secondary or vocational secondary education”<br>“Vocational training”<br>“Primary or elementary school (9th-10th grade)”<br>“Don’t know”	EFTER COVID
Healthcare worker	Registered.	Categorical variable.	“Yes”<br>“No”	EFTER COVID

Abbreviations and more descriptions here
COPD: chronic obstructive pulmonary disease
PTSD: post-traumatic stress disorder
MiBa: Danish Microbiology Database
CPR: Central Person Register
DNPR: Danish National Patient Register
EFTER COVID: After Covid Survey, a population-level survey
*BMI: Body Mass Index. Defined as BMI ≥ 30kg/m2 for individuals aged ≥18 years. For individuals aged 15-17 years, international cutoff points for obesity by sex and age were used (Cole et al.).

Cole TJ, Bellizzi MC, Flegal KM, Dietz WH. Establishing a standard definition for child overweight and obesity worldwide: international survey. Bmj. 2000;320(7244):1240.

Figure S2: Risk differences (RDs) and 95% confidence intervals (CIs) for part-time substantial sick leave one to nine months after test date between SARS-CoV-2 test-positives and negatives.

		Sick leave among		RD (95% CI)
	N	negative (n (%))	positive (n (%))	
Full population	88818	216 (0.4%)	920 (2.5%)	2.1 (2 to 2.3)
Female	57087	157 (0.5%)	758 (3.3%)	2.8 (2.6 to 3)
Male	31731	59 (0.3%)	162 (1.1%)	0.8 (0.6 to 1.1)
Older than 50 years	40439	91 (0.4%)	413 (2.6%)	2.3 (2.1 to 2.6)

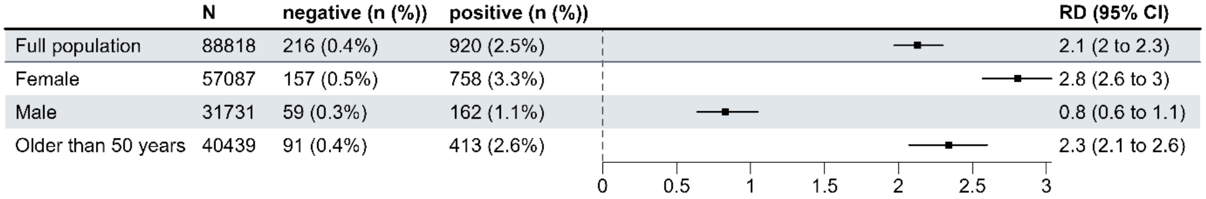

Risk differences (RDs) and 95% confidence intervals (CIs) for part-time substantial sick leave one to nine months after test date between SARS-CoV-2 test-positives and negatives. Part-time substantial sick leave is defined as >4 weeks of part-time sick leave one to nine months after the test date. Risk differences are expressed in percentage points and adjusted for age, sex, Charlson Comorbidity Index, educational level, and select pre-existing conditions (chronic diseases).

Figure S3: Risk differences (RDs) and 95% confidence intervals (CIs) for sick leave by differing duration. RDs are adjusted for age, sex, Charlson Comorbidity Index, educational level, and select pre-existing conditions (chronic diseases).

	Sick leave among		
**Length of sick leave**	**negative (n (%))**	**positive (n (%))**	**RD (95% CI)**
More than 2 weeks	1079 (2.1%)	2660 (7.1%)	5.3 (5 to 5.6)
More than 1 month (main outcome)	732 (1.4%)	1680 (4.5%)	3.3 (3 to 3.5)
More than 2 months	491 (1%)	851 (2.3%)	1.4 (1.2 to 1.6)
More than 4 months	299 (0.6%)	476 (1.3%)	0.8 (0.6 to 0.9)
More than 6 months	205 (0.4%)	310 (0.8%)	0.5 (0.4 to 0.6)
Since test date	69 (0.1%)	157 (0.4%)	0.3 (0.2 to 0.4)

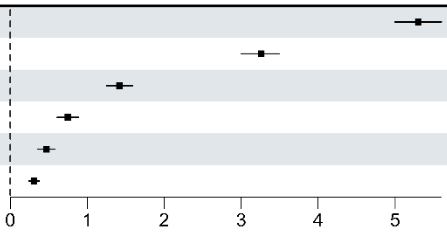

Risk differences are expressed in percentage points and adjusted for age, sex, Charlson Comorbidity Index, educational level, and select pre-existing conditions (chronic diseases).

Figure S4: A distributional plot of the underlying self-reported sick leave by age (<50 years, ≥50 years) and sex.

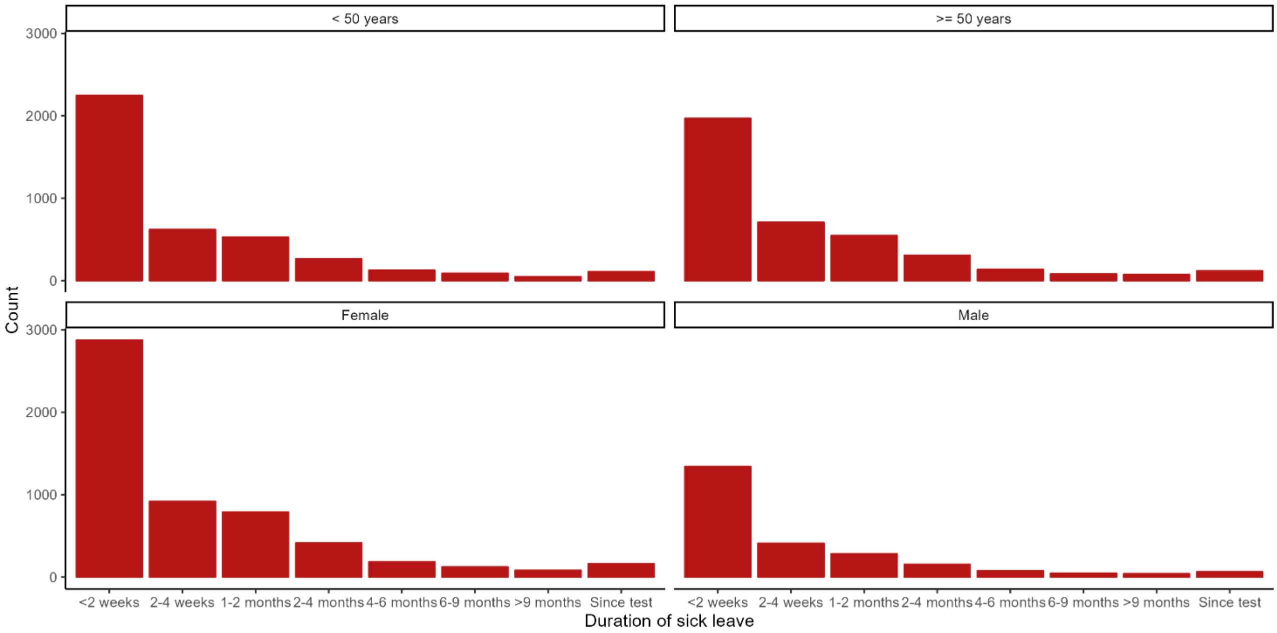

The distribution of the underlying duration of self-reported sick leave by age and sex: <50 years (upper-left), ≥50 years (upper-right), Female (lower-left), Male (lower-right).

Number of persons in each category:

N= 4023 persons <50 years

N= 3932 persons ≥ 50 years

N= 5545 females

N= 2410 males

Table S2: Risk differences (RDs) and 95% confidence intervals (CI) for substantial sick leave taken one to nine months after the test date between SARS-CoV-2 test-negatives and positives for the total study population, stratified on educational level.

**Highest completed education**	**N**	**Sick leave among test-negatives**	**Sick leave among test-positives**	**RD (95% CI)**
Primary or elementary school (9th-10th grade)	7258	48 (1.1%)	122 (4.1%)	3.7 (2.7, 4.7)
General secondary or vocational secondary education	9838	58 (1.3%)	116 (2.45)	2.1 (1.3, 2.8)
Vocational training	14245	142 (1.7%)	318 (5.5%)	3.9 (3.3, 4.6)
Higher education (1-2 years, vocational academy)	10066	81 (1.3%)	191 (4.7%)	3.1 (2.5, 3.8)
Higher education (2-4 years, BSc)	29384	272 (1.6%)	673 (5.5%)	3.8 (3.4, 4.2)
Higher education (>5 years, MSc, PhD)	15737	115 (1.2%)	207 (3.0%)	1.9 (1.4, 2.4)
Don't know	2289	16 (1.3 %)	52 (5.1%)	3.8 (2.2, 5.2)

N = 88,818 participants ages 15-65 years (response rate = 36%). $N_{positive}$ = 51,336, $N_{negative}$ = 37,482.

Risk differences are expressed in percentage points and adjusted for age, sex, Charlson Comorbidity Index, and select pre-existing conditions (chronic diseases).

Table S3: Characteristics of test-positive population divided into subcategories based on substantial sick leave and long covid hospitalization.

**Background characteristics**	**Only positive test result**	**Substantial sick leave only**	**Substantial sick leave and long covid hospitalization***	**Only long covid hospitalization**
	(N=35246)	(N=1326)	(N=354)	(N=556)
**Sex**				
Female	21390 (60.7%)	956 (72.1%)	261 (73.7%)	395 (71.0%)
Male	13856 (39.3%)	370 (27.9%)	93 (26.3%)	161 (29.0%)
**Age**				
Mean (SD)	43.3 (14.0)	48.4 (11.5)	48.6 (11.2)	46.7 (12.0)
Median [Min, Max]	46.0 [15.0, 65.0]	50.0 [15.0, 65.0]	50.0 [20.0, 65.0]	48.0 [15.0, 65.0]
**Charlson Comorbidity Index**				
0	32101 (91.1%)	1118 (84.3%)	289 (81.6%)	480 (86.3%)
1	1759 (5.0%)	118 (8.9%)	40 (11.3%)	50 (9.0%)
2	1038 (2.9%)	66 (5.0%)	18 (5.1%)	22 (4.0%)
3 or more	348 (1.0%)	24 (1.8%)	7 (2.0%)	4 (0.7%)
**Educational level (highest)**				
Higher education (>5 years, MSc, PhD)	6509 (18.5%)	173 (13.0%)	34 (9.6%)	83 (14.9%)
Higher education (2-4 years, BSc)	11235 (31.9%)	502 (37.9%)	171 (48.3%)	239 (43.0%)
Higher education (1-2 years, vocational academy)	3814 (10.8%)	159 (12.0%)	2 (9.0%)	62 (11.2%)
Primary or elementary school (9th-10th grade)	2838 (8.1%)	102 (7.7%)	20 (5.6%)	31 (5.6%)
General secondary or vocational secondary education	(12.9%)	98 (7.4%)	18 (5.1%)	37 (6.7%)
Vocational training	5343 (15.2%)	247 (18.6%)	71 (20.1%)	94 (16.9%)
Don't know	949 (2.7%)	44 (3.3%)	8 (2.3%)	10 (1.8%)
**Healthcare worker**				
No	30471 (86.5%)	1097 (82.7%)	266 (75.1%)	450 (80.9%)
Yes	4775 (13.5%)	229 (17.3%)	88 (24.9%)	88 (24.9%)
**obesity**				
no	27146 (77.0%)	850 (64.1%)	213 (60.2%)	390 (70.1%)
unknown	2507 (7.1%)	118 (8.9%)	25 (7.1%)	30 (5.4%)
yes	5593 (15.9%)	358 (27.0%)	116 (32.8%)	136 (24.5%)
**Fibromyalgia**				
No	34643 (98.3%)	1270 (95.8%)	330 (93.2%)	540 (97.1%)
Before test	285 (0.8%)	30 (2.3%)	9 (2.5%)	5 (0.9%)
**Chronic fatigue syndrome**				
No	33671 (95.5%)	1184 (89.3%)	290 (81.9%)	488 (87.8%)
Before test	434 (1.2%)	29 (2.2%)	7 (2.0%)	12 (2.2%)
**Anxiety**				
No	31495 (89.4%)	1061 (80.0%)	284 (80.2%)	461 (82.9%)
Before test	2765 (7.8%)	155 (11.7%)	21 (5.9%)	53 (9.5%)
**Depression**				

No	30449 (86.4%)	926 (69.8%)	259 (73.2%)	434 (78.1%)
Before test	3840 (10.9%)	277 (20.9%)	53 (15.0%)	79 (14.2%)
**Post traumatic stress disorder**				
No	34217 (97.1%)	1236 (93.2%)	324 (91.5%)	520 (93.5%)
Before test	636 (1.8%)	49 (3.7%)	12 (3.4%)	23 (4.1%)
**Asthma**				
No	32693 (92.8%)	1165 (87.9%)	304 (85.9%)	481 (86.5%)
Before test	2553 (7.2%)	161 (12.1%)	50 (14.1%)	75 (13.5%)
**Diabetes**				
No	34141 (96.9%)	1248 (94.1%)	330 (93.2%)	536 (96.4%)
Before test	1105 (3.1%)	78 (5.9%)	24 (6.8%)	20 (3.6%)
**High blood pressure**				
No	31678 (89.9%)	1119 (84.4%)	299 (84.5%)	493 (88.7%)
Before test	3568 (10.1%)	207 (15.6%)	55 (15.5%)	63 (11.3%)
**COPD or other lung disease**				
No	34868 (98.9%)	1295 (97.7%)	344 (97.2%)	549 (98.7%)
Before test	378 (1.1%)	31 (2.3%)	10 (2.8%)	7 (1.3%)
**Chronic or frequent headaches, including migraines**				
No	33934 (96.3%)	1227 (92.5%)	330 (93.2%)	522 (93.9%)
Before test	1312 (3.7%)	99 (7.5%)	24 (6.8%)	34 (6.1%)
*Long covid hospitalization is defined by the ICD-10 code B948A registered in the Danish National Patient Register (DNPR).				

Table S4: Background characteristics for respondents, partial-respondents, and non-respondents.

**Background characteristics**	**Completed questionnaire**	**Did not complete questionnaire**	**Partially completed questionnaire**
	(N=106917)	(N=176190)	(N=10928)
**Sex**			
Female	66161 (61.9%)	91057 (51.7%)	7004 (64.1%)
Male	40755 (38.1%)	85131 (48.3%)	3924 (35.9%)
Missing	1 (0.0%)	2 (0.0%)	0 (0%)
**Age group (years)**			
15-19	4071 (3.8%)	22922 (13.0%)	1261 (11.5%)
20-29	13000 (12.2%)	46545 (26.4%)	2073 (19.0%)
30-39	12647 (11.8%)	33914 (19.2%)	1781 (16.3%)
40-49	18969 (17.7%)	31569 (17.9%)	1861 (17.0%)
50-59	27014 (25.3%)	23935 (13.6%)	1917 (17.5%)
60-69	19551 (18.3%)	10364 (5.9%)	1134 (10.4%)
70+	11664 (10.9%)	6939 (3.9%)	901 (8.2%)
Missing	1 (0.0%)	2 (0.0%)	0 (0%)
**Charlson Comorbidity Index**			
0	92179 (86.2%)	159920 (90.8%)	9431 (86.3%)
1	7243 (6.8%)	9163 (5.2%)	760 (7.0%)
2	5293 (5.0%)	4750 (2.7%)	460 (4.2%)
3 or more	2201 (2.1%)	2355 (1.3%)	277 (2.5%)
Missing	1 (0.0%)	2 (0.0%)	0 (0%)
