# Supplementary material for: A hybrid register and questionnaire study of Covid-19 and post-acute sick leave in Denmark
Source: Nat Commun. 2023 Oct 7;14:6266. doi: 10.1038/s41467-023-42048-1 (PMC10560282; doi:10.1038/s41467-023-42048-1)
Supplement: Supplementary file 3 — Reporting Summary [file 41467_2023_42048_MOESM3_ESM.pdf]

nature portfolio

Corresponding author(s): Elisabeth O'Regan

Last updated by author(s): Aug 2, 2023

Reporting Summary

Nature Portfolio wishes to improve the reproducibility of the work that we publish. This form provides structure for consistency and transparency in reporting. For further information on Nature Portfolio policies, see our Editorial Policies and the Editorial Policy Checklist.

Statistics

For all statistical analyses, confirm that the following items are present in the figure legend, table legend, main text, or Methods section.

n/a	Confirmed	
☐	☒	The exact sample size (*n*) for each experimental group/condition, given as a discrete number and unit of measurement
☐	☒	A statement on whether measurements were taken from distinct samples or whether the same sample was measured repeatedly
☐	☒	The statistical test(s) used AND whether they are one- or two-sided<br>*Only common tests should be described solely by name; describe more complex techniques in the Methods section.*
☐	☒	A description of all covariates tested
☐	☒	A description of any assumptions or corrections, such as tests of normality and adjustment for multiple comparisons
☐	☒	A full description of the statistical parameters including central tendency (e.g. means) or other basic estimates (e.g. regression coefficient) AND variation (e.g. standard deviation) or associated estimates of uncertainty (e.g. confidence intervals)
☐	☒	For null hypothesis testing, the test statistic (e.g. *F*, *t*, *r*) with confidence intervals, effect sizes, degrees of freedom and *P* value noted<br>*Give P values as exact values whenever suitable.*
☒	☐	For Bayesian analysis, information on the choice of priors and Markov chain Monte Carlo settings
☒	☐	For hierarchical and complex designs, identification of the appropriate level for tests and full reporting of outcomes
☐	☒	Estimates of effect sizes (e.g. Cohen's *d*, Pearson's *r*), indicating how they were calculated

Our web collection on statistics for biologists contains articles on many of the points above.

Software and code

Policy information about availability of computer code

Data collection	The web-based questionnaires used for data collection were created in SurveyXact (www.surveyxact.dk). The SurveyXact system is used directly online and no version number exists.
Data analysis	All data analysis was conducted in R (version 4.2.2). The R packages "riskCommunicator" (v1.0.1) and "forestploter" (v0.2.3) was used.

For manuscripts utilizing custom algorithms or software that are central to the research but not yet described in published literature, software must be made available to editors and reviewers. We strongly encourage code deposition in a community repository (e.g. GitHub). See the Nature Portfolio guidelines for submitting code & software for further information.

Data

Policy information about availability of data

All manuscripts must include a data availability statement. This statement should provide the following information, where applicable:

- Accession codes, unique identifiers, or web links for publicly available datasets
- A description of any restrictions on data availability
- For clinical datasets or third party data, please ensure that the statement adheres to our policy

The datasets used in this study comprise sensitive, individual-level information from completed questionnaires and national register data. According to the Danish data protection legislation, the authors are not permitted to share these sensitive data directly upon request. However, the data are available for research purposes upon request to the Danish Health Authority (register data, email: kontakt@sundhedsdata.dk) and Statens Serum Institut (questionnaire data, email: aii@ssi.dk), as

well as within the framework of the Danish data protection legislation and any required permission from authorities. Data request processing can take an expected three to six months.

Human research participants

Policy information about studies involving human research participants and Sex and Gender in Research.

Reporting on sex and gender	Sex was adjusted for in the data analysis as a potential confounder and effect modifer of the effect of COVID-19 infection on substanital post-acute sick leave. Gender was not used in this study as in could not be asertained from the EFTER-COVID questionnaire. Sex was identified from the unique identifier (CPR-number) in the Danish Civil Registration System assigned to all Danish residents.
Population characteristics	The study cohort consisted of 88,818 individuals, of which 37,482 had had a confirmed SARS-CoV-2 infection. The mean age was 45 years with standard error 14 years and 64.3% were female. 62.1% had some form of higher education, while 16.0% had vocational training. The most frequent preexisting health conditions before test were high BMI (16.6%), depression (12.1%), high blood pressure (11.1%), and anxiety (8.4%).
Recruitment	All Danish residents who received a first-time positive RT-PCR test result between November 4, 2020 to February 1, 2021 and had access to the national digital communication system eboks (used by 92%) were recruited for the EFTER-COVID study. Furthermore test-negative controls were randomly selected among all Danish residents who had recieved a negative test (and no positive) using incidence density sampling on the test date with a ratio of 2:3 between test-positive and -negative persons. This ratio was chosen to compensate for a lower expected response rate among controls compared to cases. Participation bias may have occurred, where individuals living with poor health or long covid symptoms may have taken more interest in participating. Alternatively, some individuals living with long covid symptoms may have felt too poorly to participate. This could potentially lead to over- or underestiamtion of the risk of post-acute sick leave, respectively. However since the reponse rate among test-positive and test-negative are very similar, we do not believe this to be a substianl source of bias.
Ethics oversight	This study was performed as a surveillance study as part of the governmental institution Statens Serum Institut's (SSI) advisory tasks for the Danish Ministry of Health. SSI's purpose is to monitor and fight the spread of disease in accordance with section 222 of the Danish Health Act. According to Danish law, national surveillance activities carried out by SSI do not require approval from an ethics committee.

Note that full information on the approval of the study protocol must also be provided in the manuscript.

Field-specific reporting

Please select the one below that is the best fit for your research. If you are not sure, read the appropriate sections before making your selection.

☒ Life sciences ☐ Behavioural & social sciences ☐ Ecological, evolutionary & environmental sciences

For a reference copy of the document with all sections, see nature.com/documents/nr-reporting-summary-flat.pdf

Life sciences study design

All studies must disclose on these points even when the disclosure is negative.

Sample size	The sample size was not pre-determined. The final sample size is based on the number of individuals with a positive RT-PCR tests in the study period, as all these were recruited for the study and on the response rate.
Data exclusions	Participants who did not complete the questionnaire were excluded. Furthermore, we did not include individuals who indicated that they believed they previously had SARS-CoV-2 due to receiving a seropositive result for SARS-CoV-2. Participants who were >65 years were also excluded due to retirement age, where age was calculated on the test date. See Figure S1 for a detailed flowchart of our inclusion and exclusion criteria.
Replication	The present study is a questionnaire study and has not been replicated. Instead, we have compared our results to results from other sources and found them reasonably similar. An English translation of the survey has previously been made available for others to use if they wish to repeat the study.
Randomization	All persons who had recieved a positive PCR results within the study period were invited to participate. Controls were randomly selected among persons who had recieved a negative PCR result using incidence density sampling on the test date with a ratio of 2:3 between test-positive and -negative persons.
Blinding	Blinding was not relevant, since this is an observational study, where partcipants were invited based on test status (case or control).

Reporting for specific materials, systems and methods

We require information from authors about some types of materials, experimental systems and methods used in many studies. Here, indicate whether each material, system or method listed is relevant to your study. If you are not sure if a list item applies to your research, read the appropriate section before selecting a response.

Materials & experimental systems

n/a	Involved in the study
☒	☐ Antibodies
☒	☐ Eukaryotic cell lines
☒	☐ Palaeontology and archaeology
☒	☐ Animals and other organisms
☒	☐ Clinical data
☒	☐ Dual use research of concern

Methods

n/a	Involved in the study
☒	☐ ChIP-seq
☒	☐ Flow cytometry
☒	☐ MRI-based neuroimaging
