# Supplementary material for: A hybrid register and questionnaire study of Covid-19 and post-acute sick leave in Denmark
Source: Nat Commun. 2023 Oct 7;14:6266. doi: 10.1038/s41467-023-42048-1 (PMC10560282; doi:10.1038/s41467-023-42048-1)
Supplement: Supplementary file 2 — Peer Review File [file 41467_2023_42048_MOESM2_ESM.pdf]

nature portfolio

Peer Review File

A hybrid register and questionnaire study of Covid-19 and post-acute sick leave in Denmark

Open Access This file is licensed under a Creative Commons Attribution 4.0 International License, which permits use, sharing, adaptation, distribution and reproduction in any medium or format, as long as you give appropriate credit to the original author(s) and the source, provide a link to the Creative Commons license, and indicate if changes were made. In the cases where the authors are anonymous, such as is the case for the reports of anonymous peer reviewers, author attribution should be to 'Anonymous Referee' followed by a clear attribution to the source work. The images or other third party material in this file are included in the article's Creative Commons license, unless indicated otherwise in a credit line to the material. If material is not included in the article's Creative Commons license and your intended use is not permitted by statutory regulation or exceeds the permitted use, you will need to obtain permission directly from the copyright holder. To view a copy of this license, visit http://creativecommons.org/licenses/by/4.0/.

REVIEWER COMMENTS

Reviewer #1 (Remarks to the Author):

This study uses a combination of routine health register and questionnaire survey data to establish pattens of sick leave from work following COVID-19 infection. The key result is of far higher proportion of people taking ¬> 1 month sick leave if they'd had a positive SARS-CoV-2 test compared to negative test, with an absolute difference of 3%. If valid and reflected elsewhere this is a measure of the economic as well as the personal health impact of the pandemic. It should be emphasized that this is in an almost entirely unvaccinated population (i.e. the infections occurred between November 2020 and Feb 2021). Results cannot be extrapolated to cover later variants and vaccinated populations, and therefore the work feels out of date.

The report is interesting and adds a population level perspective, while other studies are often based on selected and biased sub-populations, for example those who attend healthcare with post COVID conditions. The population sampling frame, and the test-negative control group are strengths of the study. The work is based on self-reported sick leave, although it seems unlikely that this is biased by prior COVID test result. There is no exploration of the potential biases introduced by differential participation – I suggest that a supplementary table is added comparing respondents with non-respondents for available data (age, sex and comorbidities?)

The discussion raises the suggestion that sick leave is a proxy for Long COVID. While this may be indicative of some of the longer term sequelae of infection, not all of these are included in the definition of long COVID. An increase in some cardiovascular and metabolic conditions following infection has been described in a number of studies, but including these in the umbrella term of Long COVID is questionable. Only 20% of these people had a diagnosis of Long COVID, it would be useful to know the diagnoses for the other 80%, and to relate these to pre-COVID conditions.

The methods indicate that sick leave is widely available in Denmark, which also limits the applicability of the findings and methods to other countries. It would also be useful to know if the diagnosis of Long COVID leads to any disability payments. The authors recognise the limitation of not having occupational data, and there is no pre-COVID sick leave data. This is a shame, because it is possible that those who work in manual and less flexible roles are less able to take time off at the time of initial illness, and this has been associated with poorer outcomes in some studies.

There is mention of a stakeholder group that provided input into the underlying EFTER-COVID study; were the public participants in this group? I think this is now good practice in research.

Reviewer #2 (Remarks to the Author):

Covid-19 and post-acute sick leave: a hybrid register and questionnaire study in the adult Danish population

General comments and summary of recommendation:

The article “Covid-19 and post-acute sick leave: a hybrid register and questionnaire study in the adult Danish population” examines the impacts of long COVID on self-reported sick leave of greater than 1 month in individuals during the index/alpha waves of the COVID-19 pandemic within the 1-9 months period after being tested positive, in the Danish population.

MAJOR COMMENTS:

Out of all the test-positives, 1.6% did not take substantial sick leave. A systematic analysis of their risk factor, co-morbidities is needed here. Mainly comparing the individuals who tested positive with substantial sick leave vs. the ones without the substantial sick leave.

Repeat infections with SARS-CoV-2 could be one of drivers of long COVID symptoms - Is this phenomenon accounted for patients, who got infected and took extended sick leave.

Authors have not determined the impact of working ability of the individuals i.e ability to perform the essential functions of the job when they return back to work from an extended sick leave - compared to the controls as the question of vocational training has been asked in the survey.

Less than 1% of participants had received one or more doses of a vaccine against Covid-19. Please provide further analysis of this subset of patients who have received the single dose or both doses of COVID vaccine. Is 1.6% of COVID + patients “without” substantial sick leave are part of this group?

Prevalence of the fibromyalgia is low 0.9% in the study population that might have contributed to a more pronounced effect in the model. What is the prevalence of fibromyalgia among the Danish population? Is there a higher prevalence of fibromyalgia in women and women over 50 years of age in the Danish population?

It is unclear from the description as to why the healthcare worker population was included in the survey. We probably need to define the “healthcare” workers with the risk differences (RD) of 4.6 than the general population. Why are healthcare workers chosen as a subset either due to exposure, convenient sampling, survey response, etc?

For part-time sick leave, individuals ≥50 years had a similar risk compared to that of the full study population. Can we further explain the unique characteristics of this subset of individuals?

What is an index and alpha waves? Please describe in detail the index vs. alpha waves. How it was determined if the circulating virus was index or alpha (B.1.1.7) virus strain. Whether the SARS-CoV-2 genomic sequencing was done as part of the genomic surveillance?

Authors point toward the vaccination and omicron variant but the alpha variant preceded the beta (B.1.351) and delta variant (AY.4.2), both had profound impact on the long covid symptoms in the subset of population infected with the variants. Are we accounting for the beta and delta variant in the study?

Exploring the incentive provided for taking the sick leave - it seems to be a generous system with full pay being provided during the sick leave. Most individuals might not have not participated in the study if they might be able to exploit the system with self reported COVID-19 symptoms or too afraid to return to work for potentially getting infected by the deadly virus.

As noted “self-reported sick leave was not necessarily taken consecutively such that it could capture multiple periods of sick leave due to fluctuating symptoms” Is there more information on the reason behind taking the extended sick leave? Are we able to differentiate between the proportion of the sick leave, not related to the COVID symptoms - common in individuals with co-morbidities like Diabetes Mellitus type II, COPD, fibromyalgia, depression. They might have experienced good days or bad days depending on their disease status. One is able to take sick leave for a myriad of reasons.

In terms of co-morbidities, were the immunocompromised status or being on steroids considered for the infected group vs. the non-infected group?

Are we able to consider extended sick leaves for individuals who not only got infected with the virus but stayed home to address the needs of a positive family member or a child.

MINOR COMMENTS:

Please include the survey questionnaire in the supplement

Describe the “fluctuating illness” - need a clear understanding of what “fluctuating illness” means in the terms of long covid symptoms.

Unable to find the RD for healthcare workers vs. the general population.

Reviewer #3 (Remarks to the Author):

In this article, the authors tried to establish substantial sick leave as a proxy for long covid and identify the risk factors for substantial sick leave. The main part of statistical analysis is logistic regression based

on Danish national data of both test-positive and test-negative people. However, I have major reservations about their claims and results, which are listed below.

Major:

1. More statistical analysis and evidence are needed to claim sick leave as a proxy for long covid. In the first paragraph of Discussion, the authors stated that “20.1% of the test-positives who took substantial sick leave also had a registered ICD-10 code for long covid.” In my opinion, this is not enough to support the claim, as it also means that most of test-positives who took substantial sick leave did not have a ICD-10 code for long-covid. Detailed analysis based on data, such as contingency table, receiver operating characteristic (ROC) curve and area under curve (AUC), are required to reveal the actual relations between substantial sick leave and long covid. Such evidence is crucial because this claim is the foundation of the whole article.

2. Using the sick leave as a proxy for long covid has another major drawback. As the authors pointed out in the Figure 1, “N = 88,818 participants ages 15-65 were included”, only working age population were included in the study. However, patients over the age of 65 account for a large proportion of COVID-19 deaths and disease burden. Therefore, a proxy that can be representative for all ages is of greater social and public health significance and should be considered.

3. Risk difference for the full population is 3.3 (95% CI: 3.0 to 3.5) in the Figure 3, and almost all the risk groups with clinical characteristics before test date have a risk difference no smaller than 3.3. Are these results subject to Simpson’s paradox in statistics? I suggest the authors could perform some analysis on the possible confounders. Or at least, they should give the results for the group with no pre-existing condition.

Minor:

1. In the Abstract, the authors stated that “certain pre-existing conditions such as fibromyalgia increased risks for taking substantial sick leave”, while in the first paragraph of Discussion, they claimed “obesity and a variety of pre-existing health conditions as possible risk factors”. This is inconsistent and could be confusing for the readers.

2. N = 88,818 participants ages 15-65 were included in the study, but one of the age groups showed in the Figure 2C is 60-69.

Reviewer #1 (Remarks to the Author):

This study uses a combination of routine health register and questionnaire survey data to establish pattens of sick leave from work following COVID-19 infection. The key result is of far higher proportion of people taking ¬> 1 month sick leave if they'd had a positive SARS-CoV-2 test compared to negative test, with an absolute difference of 3%. If valid and reflected elsewhere this is a measure of the economic as well as the personal health impact of the pandemic. It should be emphasized that this is in an almost entirely unvaccinated population (i.e. the infections occurred between November 2020 and Feb 2021).**(1)** Results cannot be extrapolated to cover later variants and vaccinated populations, and therefore the work feels out of date.**(2)**

The report is interesting and adds a population level perspective, while other studies are often based on selected and biased sub-populations, for example those who attend healthcare with post COVID conditions. The population sampling frame, and the test-negative control group are strengths of the study. The work is based on self-reported sick leave, although it seems unlikely that this is biased by prior COVID test result. There is no exploration of the potential biases introduced by differential participation – I suggest that a supplementary table is added comparing respondents with non-respondents for available data (age, sex and comorbidities?) **(3)**

The discussion raises the suggestion that sick leave is a proxy for Long COVID. While this may be indicative of some of the longerterm sequelae of infection, not all of these are included in the definition of long COVID. An increase in some cardiovascular and metabolic conditions following infection has been described in a number of studies, but including these in the umbrella term of Long COVID is questionable. **(4)** Only 20% of these people had a diagnosis of Long COVID, it would be useful to know the diagnoses for the other 80%, and to relate these to pre-COVID conditions**. (5)**

The methods indicate that sick leave is widely available in Denmark, which also limits the applicability of the findings and methods to other countries. It would also be useful to know if the diagnosis of Long COVID leads to any disability payments. **(6)** The authors recognise the limitation of not having occupational data, and there is no pre-COVID sick leave data. This is a shame, because it is possible that those who work in manual and less flexible roles are less able to take time off at the time of initial illness, and this has been associated with poorer outcomes in some studies.

There is mention of a stakeholder group that provided input into the underlying EFTER-COVID study; were the public participants in this group? **(7)** I think this is now good practice in research.

Response to Reviewer 1

We thank Reviewer 1 for their careful consideration of our manuscript and are pleased to reflect on their comments.

(1) We agree that it is important to emphasize the period of study/when infections occurred and have tried to make clear how this limits the generalizability of our study findings. We think that these additions will help make this even more transparent to readers.

We have now further emphasized the study period in the introduction by adding the following: "Here, we examine post-acute sick leave following infections which took place during the index- and alpha-dominant periods in Denmark by comparing persons infected with SARS-CoV-2 to persons with no known history of SARS-CoV-2 infection."

In the discussion, we also reflect on the limitations of our findings with respect to the study period in question:

"Lastly, our results capture index- and alpha-variant infections which largely occurred before SARS-CoV-2 vaccine rollout and we cannot exclude that the absolute magnitude of our results are attenuated by vaccinations and the omicron variant."

In our conclusion (the final paragraph of the discussion), we have also stated the study period in the first sentence:

"Among individuals infected with SARS-CoV-2 during the index and alpha waves, an additional 33 individuals per 1000 took substantial sick leave within 1-9 months following acute infection compared to persons with no known infection."

(2) This study presents retrospective analyses underscoring the burden of post-acute sick leave after SARS-CoV-2 infection and highlights special risk groups for sick leave during the index- and alpha-dominant periods, which has otherwise not been evaluated using a study design with test-negative controls. It is important for researchers to explore how this burden compares following different advents in the pandemic, such as mass-vaccination, different variants, and reinfection. Our study, together with others which have used longitudinal data, are needed to collectively gain a detailed understanding of the evolving, post-acute burden of Covid-19 on society.

To help bring this point forward, we have substantially revised our introduction and discussion.

(3) We have now added a supplementary Table (Table S4) to show the register-based background characteristics (sex, age, Charlson Comorbidity Index) for respondents, partial-respondents, and non-respondents. Respondents were more likely to be female, older (50-65 years), and have a slightly higher Charlson Comorbidity Index. In the Results section, under Overview of study population, we have added the following:

“Respondents were more likely to be female, older (50-65 years), and have a higher Charlson Comorbidity Index than non-respondents (Table S4)”.

(4) To clarify, our discussion was formulated to highlight what previous literature has noted with respect to possible risk factors for longer-term sick leave following infection with SARS-CoV-2. Thus, the main focus is not on new-onset conditions following infection but rather exploring conditions which preceded the infection which might increase the risk of post-acute substantial sick leave. Furthermore, as described above, we no longer frame post-acute sick leave as a proxy measure of long covid burden.

(5) To elaborate on the point above (4), self-reported pre-existing conditions and Charlson Comorbidity Index at the test-date for persons who tested positive and took substantial sick leave but did NOT have a registered long covid hospitalization is available in Table S3, column 3.

(6) Payments for sick leave are not contingent on having a registered disability in Denmark. There are no specific disability payments for having a long covid diagnosis.

(7) The EFTER-COVID group has encouraged patient participation where possible. The inclusion of post-exertional malaise questions in the fatigue questionnaire (one of several questionnaire tracks in EFTER-COVID) was a result of a ”myalgic encephalomyelitis/chronic fatigue syndrome”-patient advocate directly contacting Anders Hviid, and on March 13, 2023, Statens Serum Institut (the host institution of EFTER-COVID) held a town hall meeting on long covid open to the public. A large proportion of the attendees were patients suffering from post-acute symptoms and a representative from a newly formed long covid patient advocate group was also invited to speak.

End of responses to Reviewer 1

Reviewer #2 (Remarks to the Author):

Covid-19 and post-acute sick leave: a hybrid register and questionnaire study in the adult Danish population

General comments and summary of recommendation:

The article "Covid-19 and post-acute sick leave: a hybrid register and questionnaire study in the

adult Danish population" examines the impacts of long COVID on self-reported sick leave of greater than 1 month in individuals during the index/alpha waves of the COVID-19 pandemic within the 1-9 months period after being tested positive, in the Danish population.

MAJOR COMMENTS:

Out of all the test-positives, 1.6% did not take substantial sick leave. **(1)** A systematic analysis of their risk factor, co-morbidities is needed here. Mainly comparing the individuals who tested positive with substantial sick leave vs. the ones without the substantial sick leave. **(2)**

Repeat infections with SARS-CoV-2 could be one of drivers of long COVID symptoms - Is this phenomenon accounted for patients, who got infected and took extended sick leave. **(3)**

Authors have not determined the impact of working ability of the individuals i.e., ability to perform the essential functions of the job when they return back to work from an extended sick leave - compared to the controls as the question of vocational training has been asked in the survey.

Less than 1% of participants had received one or more doses of a vaccine against Covid-19. Please provide further analysis of this subset of patients who have received the single dose or both doses of COVID vaccine. Is 1.6% of COVID + patients "without" substantial sick leave are part of this group? **(4)**

Prevalence of the fibromyalgia is low 0.9% in the study population that might have contributed to a more pronounced effect in the model. What is the prevalence of fibromyalgia among the Danish population? Is there a higher prevalence of fibromyalgia in women and women over 50 years of age in the Danish population? **(5)**

It is unclear from the description as to why the healthcare worker population was included in the survey. We probably need to define the "healthcare" workers with the risk differences (RD) of 4.6 than the general population. Why are healthcare workers chosen as a subset either due to exposure, convenient sampling, survey response, etc? **(6)**

For part-time sick leave, individuals ≥50 years had a similar risk compared to that of the full study population. Can we further explain the unique characteristics of this subset of individuals? **(7)**

What is an index and alpha waves? Please describe in detail the index vs. alpha waves. How it was determined if the circulating virus was index or alpha (B.1.1.7) virus strain. Whether the SARS-CoV-2 genomic sequencing was done as part of the genomic surveillance? **(8)**

Authors point toward the vaccination and omicron variant but the alpha variant preceded the beta (B.1.351) and delta variant (AY.4.2), both had profound impact on the long covid symptoms in the subset of population infected with the variants. Are we accounting for the beta and delta variant in the study? **(9)**

Exploring the incentive provided for taking the sick leave - it seems to be a generous system with full pay being provided during the sick leave. Most individuals might not have not participated in the study if they might be able to exploit the system with self reported COVID-19 symptoms or too afraid to return to work for potentially getting infected by the deadly virus.

As noted "self-reported sick leave was not necessarily taken consecutively such that it could capture multiple periods of sick leave due to fluctuating symptoms" Is there more information on the reason behind taking the extended sick leave? Are we able to differentiate between the proportion of the sick leave, not related to the COVID symptoms - common in individuals with co-morbidities like Diabetes Mellitus type II, COPD, fibromyalgia, depression. They might have experienced good days or bad days depending on their disease status. One is able to take sick leave for a myriad of reasons. **(10)**

In terms of co-morbidities, were the immunocompromised status or being on steroids considered for the infected group vs. the non-infected group? **(11)**

Are we able to consider extended sick leaves for individuals who not only got infected with the virus but stayed home to address the needs of a positive family member or a child. **(12)**

MINOR COMMENTS:

(1) Please include the survey questionnaire in the supplement

(2) Describe the "fluctuating illness" - need a clear understanding of what "fluctuating illness" means in the terms of long covid symptoms.

(3) Unable to find the RD for healthcare workers vs. the general population.

Response to Reviewer 2

We thank the reviewer for their comments on our manuscript, and we appreciate the opportunity to respond.

Major comments

(1) We understand that the way this section was written before could have been formulated more clearly.

To clarify, out of all the test-positives who took substantial sick leave, 21.1% (N = 354) also had a hospital-registered long covid diagnosis. However, out of all the test-positives who did not take substantial sick leave, only 1.6% (N = 556) also had a hospital-registered long covid diagnosis. These numbers demonstrate that the proportion of individuals with a long covid diagnosis among test-positives with substantial sick leave was substantially greater than among test-positives who did not take substantial sick leave.

We have now modified the subsection "Prevalence of substantial sick leave" under Results to read as follows: "Notably, out of all test-positives who took substantial sick leave, 21.1% (N = 354) had received a diagnosis with sequelae of SARS-CoV-2 (ICD-10 code B948A) (Figure 2, Panel A). However, out of all the test-positives who did not take substantial sick leave, only 1.6% (N = 556) also had a hospital-registered diagnosis with sequelae of SARS-CoV-2."

(2) Please see our response to (1). A comparison between individuals who tested positive with and without substantial sick leave is outlined in Table S3. Test-positives with substantial sick leave are more likely to be females, older, and have more underlying conditions. The focus of this paper is drawing comparison between test-positives and test-negatives to understand the attributable risk of SARS-CoV-2 infection on substantial sick leave, not to understand differences between test-positives who took substantial sick leave and who did not. We want to emphasize, that in this comparison, we do take potential confounders, such as sex, age and comorbidities, into account.

(3) People were not invited to fill out this questionnaire if they were re-infected with SARS-CoV-2.

(4) Please see the response in (1) which reflects what this 1.6% refers to. As indicated in our main results (Figure 3), it is 4.5% of all test-positives included in the study who took substantial sick leave. The focus of this study is not to investigate the impact of vaccination on sick leave, although much literature suggests that vaccination might be protective of long covid symptoms (https://bmjmedicine.bmj.com/content/2/1/e000385) and thus could also protect against post-acute sick leave.

It is important to note that <1% of our study sample was vaccinated, and those who were during this period represent a special group of individuals (e.g., high risk or healthcare worker). Fewer than 400 individuals were vaccinated (and this was only partial vaccination) prior to responding to the questionnaire. With fewer than 20 vaccinated people taking substantial sick leave (both test-positives and test-negatives), we do not think that this should influence our findings, and we are furthermore unable to assess the relationship between (partial) vaccination and substantial sick leave. The crude risk for substantial sick leave for vaccinated, test-positives was 5.2%, whereas the crude risk for substantial sick leave for vaccinated, test-negatives was 2.2%.

(5) An estimate from 1993 suggested that the prevalence of fibromyalgia in the Danish population between 18 and 79 years of age was 0.66% (95% CI 0.28%-1.29%). This study also noted that that significantly more females than males met inclusion criteria, and that persons with fibromyalgia tended to be older (https://pubmed.ncbi.nlm.nih.gov/8235493/ https://pubmed.ncbi.nlm.nih.gov/8235493/). The prevalence of fibromyalgia in our study compares well with the aforementioned study.**(6)** We were able to identify healthcare workers through work authorizations in the Danish Register of Healthcare Professionals and have now made this clearer in-text under Online Methods, Data sources, as well as elaborated more on the higher risk among healthcare workers in the Discussion:

Data sources: "…healthcare workers from work authorization from the Danish Register of Healthcare Professionals"

Discussion: "While we observed that healthcare workers had a greater risk difference for post-acute substantial sick leave than the general population, little is known about the post-acute burden of Covid-19 infection in healthcare workers. Healthcare workers are at an elevated risk of acute Covid-19 infection compared to the general population due to greater exposure, and as such, the burden of post-acute sick leave may be larger (Al-Oraibi, 2022)."

(7) We have rephrased this sentence to be clearer: "However, with regards to individuals aged 50-65 years, a greater difference in RD was noted for full-time sick leave than for part-time sick leave." The reason for why persons in this age group are more likely to take full-time sick leave rather than part-time sick leave can be speculated, but it is probably due to older individuals having more comorbidities and thus experiencing greater illness from infection.

(8) In Denmark, variant dominance was determined through extensive, nationwide whole genome sequencing:
https://www.sciencedirect.com/science/article/pii/S2666776222001466?ref=pdf_download&fr=RR-2&rr=7e912542ae46be53,https://www.eurosurveillance.org/content/10.2807/1560-7917.ES.2021.26.50.2101146?crawler=true) . We did not have the variant data in this study, but we refer to the period infections occurred and which variants were dominant during this period (index and alpha).

We have now specified this under Online Methods, Study context, Denmark's SARS-CoV-2 testing strategy:

"…periods of variant dominance has been determined by extensive whole genome sequencing (Michlmayr et al., 2022)."

(9) Please see the point above concerning how we identified which variants were dominant during this period of infections. We did not examine infections which took place during Beta or Delta-dominant waves.

(10) The questionnaire did not ask the reasons for why the sick leave was taken. Given that we compare test-positives to test-negatives both in the general study population and within patient groups with adjustment for potential confounders (risk differences presented in figure 3), and still see significant risk differences, one can infer that the difference in sick leave is attributable to SARS-CoV-2 infection (to the degree that unmeasured confounding can be ruled out).

(11) The list of possible risk groups we explored are listed under Methods:

"To investigate possible risk groups for substantial sick leave following infection with SARS-CoV-2, we conducted analyses on sub-populations defined by possible risk factors. These risk factors were defined apriori based on available variables in the survey. These included middle to older age (categorized as ≥50 years) , female sex, obesity, diabetes, asthma, high blood pressure, COPD or other chronic lung disease, chronic or frequent headaches/migraines, and the following health conditions diagnosed by a medical doctor before the test: depression, anxiety, post-traumatic stress disorder, chronic fatigue syndrome, and fibromyalgia."

We did not examine persons undergoing steroid treatment. Certain comorbid conditions which classify people as "immunocompromised" are considered, as we adjust for Charlson Comorbidity Index, which includes AIDS, leukemia, liver disease, amongst other conditions.

(12) Although this would be interesting to look at, we unfortunately do not have data on childrens' sick leave.

Minor comments

(1) The English translation of the questionnaire is available online: https://static-content.springer.com/esm/art%3A10.1038%2Fs41467-022-31897-x/MediaObjects/41467_2022_31897_MOESM1_ESM.pdf, pg. 11. We have now referenced it in-text: An English translation of the questionnaire is available in the supplementary materials of a previous EFTER-COVID study (Sørensen et al. 2022).

(2) The World Health Organization specifies the following in the clinical case definition of post COVID-19 condition:

"Symptoms may be new onset, following initial recovery from an acute COVID-19 episode or persist from the initial illness. Symptoms may also fluctuate or relapse over time." (https://www.who.int/teams/health-care-readiness/post-covid-19-condition)

(3) Under Results, Risk differences for substantial sick leave, we have written the following:

"Healthcare workers (N=13,872) also had a larger risk difference (RD 4.6, 95% CI 3.9 to 5.3) than the general population (RD 3.3, 95% CI 3.0 to 3.5)."

End of responses to Reviewer 2

Reviewer #3 (Remarks to the Author):

In this article, the authors tried to establish substantial sick leave as a proxy for long covid and identify the risk factors for substantial sick leave. The main part of statistical analysis is logistic regression based on Danish national data of both test-positive and test-negative people. However, I have major reservations about their claims and results, which are listed below.

Major:

(1) More statistical analysis and evidence are needed to claim sick leave as a proxy for long covid. In the first paragraph of Discussion, the authors stated that "20.1% of the test-positives who took substantial sick leave also had a registered ICD-10 code for long covid." In my opinion, this is not enough to support the claim, as it also means that most of test-positives who took substantial sick leave did not have a ICD-10 code for long-covid. Detailed analysis based on data, such as contingency table, receiver operating characteristic (ROC) curve and area under curve (AUC), are required to reveal the actual relations between substantial sick leave and long covid. Such evidence is crucial because this claim is the foundation of the whole article.

(2) Using the sick leave as a proxy for long covid has another major drawback. As the authors pointed out in the Figure 1, "N = 88,818 participants ages 15-65 were included", only working age population were included in the study. However, patients over the age of 65 account for a large proportion of COVID-19 deaths and disease burden. Therefore, a proxy that can be representative for all ages is of greater social and public health significance and should be considered.

(3) Risk difference for the full population is 3.3 (95% CI: 3.0 to 3.5) in the Figure 3, and almost all the risk groups with clinical characteristics before test date have a risk difference no smaller than 3.3. Are these results subject to Simpson's paradox in statistics? I suggest the authors could perform some analysis on the possible confounders. Or at least, they should give the results for the group with no pre-existing condition.

Minor:

(1) In the Abstract, the authors stated that "certain pre-existing conditions such as fibromyalgia increased risks for taking substantial sick leave", while in the first paragraph of Discussion, they claimed "obesity and a variety of pre-existing health conditions as possible risk factors". This is inconsistent and could be confusing for the readers.

(2) N = 88,818 participants ages 15-65 were included in the study, but one of the age groups showed in the Figure 2C is 60-69.

Response to Reviewer 3

We kindly thank Reviewer 3 for their careful consideration of the manuscript and value the opportunity to improve the paper in-line with their comments.

RESPONSES TO MAJOR COMMENTS

(1) We have substantially revised the introduction and discussion sections of our manuscript to reframe post-acute sick leave as one of the societal consequences of SARS-COV-2 infection rather than as a proxy for long covid. We have not carried out the suggested analysis as we have substantially reformulated the paper to align with feedback from all reviewers and the editor, such that the paper describes post-acute sick leave as a prolonged consequence of SARS-CoV-2 infection rather than a proxy for long covid.

(2) We understand the concern that post-acute sick leave cannot be used as a measure of the societal burden among older (retired) populations, and have now reflected on this important point under our limitations section: "Finally, post-acute sick leave is not a suitable measure for retired or non-working individuals, and as such, other indicators of the post-acute burden of SARS-CoV-2 infection are needed."

(3) Simpson's paradox occurs when the relationship between variables changes/reverses when combining the subgroups, leading to a different conclusion than when the subgroups are analyzed separately. Hence, to test if Simpson's paradox is present, we computed the difference in risk of substantial sick leave between test-positives and -negatives on the *combined* data of the subgroups with pre-existing conditions before test date (N = 38,067). This yielded the same conclusion (RD 4.6, 95% CI 4.1 to 5.0) with a risk difference higher than that in the full population. Furthermore, we carried out an analysis on the group with no self-reported comorbidities and a Charlson comorbidity score of 0 (N = 47,916). Here, we observed a smaller risk difference (RD 2.2, 95% CI 2.0 to 2.5) than that of the full study population.

Therefore, given the consistency of the results across subgroups and the absence of any contradictory findings when combining the data, we do not believe that Simpson's paradox is present in our study. Conversely, the consistent findings across the pre-existing conditions suggest that people with pre-existing conditions, who are infected with SARS-CoV 2 were at a greater increased risk of taking substantial sick leave compared to people with the same pre-existing conditions but with no known history of infection.

RESPONSES TO MINOR COMMENTS

(1) We have now made all of the highlighted risk factors consistent across sections.

(2) We have now corrected this error in Figure 2C.

End of responses to Reviewer 3

REVIEWERS' COMMENTS

Reviewer #1 (Remarks to the Author):

I am happy that the authors have addressed the points in my review and those of the other reviewers. As a result the article is more clear about the findings and limitations.

Thank you for asking me to re-review.

Reviewer #2 (Remarks to the Author):

Thank you so much for the response to all the comments. The authors have made appropriate changes in the manuscript based on the comments/recommendations.
